# 1337. Detection of SARS-CoV-2 using an electrostatic precipitator air sampler in hospital patient rooms of coronavirus disease-2019 and public areas

**DOI:** 10.1093/ofid/ofad500.1174

**Published:** 2023-11-27

**Authors:** Hiroaki Baba, Kyohei Fukuda, Mie Yoshida, Kouichi Kitabayashi, Shinjirou Katsushima, Hiroki Sonehara, Kazue Mizuno, Hajime Kanamori, Koichi Tokuda, Atsuhiro Nakagawa, Akira Mizuno

**Affiliations:** Tohoku University Graduate School of Medicine, Sendai, Miyagi, Japan; AMANO Co., Ltd., Yokohama, Kanagawa, Japan; AMANO Co., Ltd., Yokohama, Kanagawa, Japan; AMANO Co., Ltd., Yokohama, Kanagawa, Japan; AMANO Co., Ltd., Yokohama, Kanagawa, Japan; Genome Clinic Co., Ltd., Chiba, Chiba, Japan; Preferred Networks, Inc., Chiyoda, Tokyo, Japan; Tohoku University Graduate School of Medicine, Sendai, Miyagi, Japan; Tohoku University Graduate School of Medicine, Sendai, Miyagi, Japan; Tohoku University Graduate School of Medicine, Sendai, Miyagi, Japan; Amano Corporation, Nagoya, Aichi, Japan

## Abstract

**Background:**

Monitoring of airborne viruses in the air is a crucial step in the design of appropriate prevention and control measures. We used a novel wet-type electrostatic precipitator air sampler using a viral dissolution buffer containing a radical scavenging agent to detect severe acute respiratory syndrome coronavirus 2 (SARS-CoV-2) RNA in the air of a hospital room of coronavirus disease-2019 (COVID-19) patients and public areas.

**Methods:**

The concentration of SARS-CoV-2 RNA in the air of hospital rooms of a total of 10 consented patients hospitalized for COVID-19 was measured using an electrostatic precipitator air sampler between February and August 2022. The air sampler was also used to measure the concentration of SARS-CoV-2 RNA in the air of an office room during the working hour, a food court of a shopping mall during lunchtime on a holiday, and a station corridor during morning and evening commuting hours. The office room and food court were 340 m^3^ and 2000 m^3^, respectively, and contained about 30 and 300 people, respectively, who removed their masks when eating, drinking, and talking. The station corridor was 1500 m^3^ in size and contained 100 people all wearing masks and had been walking during sampling. SARS-CoV-2 RNA collected in Buffer AVL was extracted using QIAamp Viral RNA mini kit and quantified by the real-time quantitative PCR (RT-qPCR) targeting the N2 region. SARS-CoV-2 RNA concentration in the air of each space was calculated from the amount of collected viral RNA, the air volume required for the collection, and the particle collection efficiency of the sampler.

**Results:**

SARS-CoV-2 RNA ranging from 1.0 x 10^2^ to 2.4 x 10^4^ copies/m^3^ was detected in the air of the rooms of all eligible patients (Table 1). The patient with the longest viral detection in the air of the room was a severely ill patient with 1.3 x 10^3^ copies/m^3^ viral RNA until 18 days after illness onset. In the office room and food court, 7.8 x 10^2^ copies/m^3^ and 1.9 x 10^2^ copies/m^3^ viral RNA were detected, respectively (Table 2).

**Table 1.**
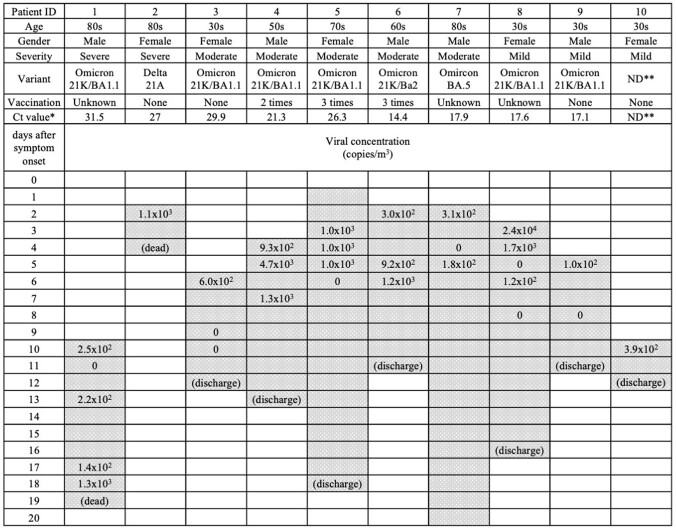
Clinical characteristics of the patients and SARS-CoV-2 viral RNA concentrations in the hospital room air.

The Gray shaded columns indicate the hospitalization period. *Ct value: Cycle threshold values of nasopharyngeal SARS-CoV-2 PCR on admission. ND**: Not detected.

**Table 2.**
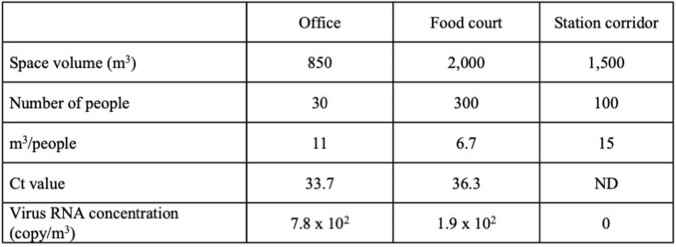
RT-qPCR detection results of SARS-CoV-2 in the indoor environment and suspended concentrations.

**Conclusion:**

The electrostatic precipitator air sampler is useful for measuring SARS-CoV-2 RNA in the air of hospital rooms of COVID-19 patients and of public spaces. Airborne viral RNA monitoring may aid in appropriate airborne infection control.

**Disclosures:**

**Hiroaki Baba, MD, PhD**, Amano Co., Ltd.: Grant/Research Support **Kyohei Fukuda, n/a**, AMANO Co., Ltd.: KF was supported by AMANO Co., Ltd. in the form of salaries. **Mie Yoshida, n/a**, AMANO Co., Ltd.: MY was supported by AMANO Co., Ltd. in the form of salaries. **Kouichi Kitabayashi, n/a**, AMANO Co., Ltd.: KK was supported by AMANO Co., Ltd. in the form of salaries. **Shinjirou Katsushima, n/a**, AMANO Co., Ltd.: SK was supported by AMANO Co., Ltd. in the form of salaries. **Hajime Kanamori, MD, PhD, MPH**, Amano Co., Ltd.: Grant/Research Support **Koichi Tokuda, MD, PhD, MPH**, AMANO Co., Ltd.: Grant/Research Support

